# Feeling Younger on Active Summer Days? On the Interplay of Behavioral and Environmental Factors With Day-to-Day Variability in Subjective Age

**DOI:** 10.1093/geroni/igae067

**Published:** 2024-07-17

**Authors:** Laura I Schmidt, Fiona S Rupprecht, Martina Gabrian, Carl-Philipp Jansen, Monika Sieverding, Hans-Werner Wahl

**Affiliations:** Institute of Psychology, Heidelberg University, Heidelberg, Germany; Department of Developmental and Educational Psychology, University of Vienna, Vienna, Austria; Institute of Psychology, Heidelberg University, Heidelberg, Germany; Robert Bosch Hospital, Clinic for Geriatric Rehabilitation, Stuttgart, Germany; Geriatric Center, Heidelberg University Hospital, Heidelberg, Germany; Institute of Psychology, Heidelberg University, Heidelberg, Germany; Institute of Psychology, Heidelberg University, Heidelberg, Germany; Network Aging Research (NAR), Heidelberg University, Heidelberg, Germany

**Keywords:** Felt age, Physical activity, Sleep quality, Views on aging, Weather conditions

## Abstract

**Background and Objectives:**

Subjective age, that is, how old people feel in relation to their chronological age, has mostly been investigated from a macro-longitudinal, lifespan point of view and in relation to major developmental outcomes. Recent evidence also shows considerable intraindividual variations in micro-longitudinal studies as well as relations to everyday psychological correlates such as stress or affect, but findings on the interplay with physical activity or sleep as behavioral factors and environmental factors such as weather conditions are scarce.

**Research Design and Methods:**

We examined data from 80 recently retired individuals aged 59–76 years (*M* = 67.03 years, 59% women) observed across 21 days. Daily diary-based assessments of subjective age, stress, affect, and sleep quality alongside physical activity measurement via Fitbit (steps, moderate-to-vigorous physical activity) and daily hours of sunshine were collected and analyzed using multilevel modeling.

**Results:**

Forty-four percent of the overall variance in subjective age was due to intraindividual variation, demonstrating considerable fluctuation. Affect explained the largest share in day-to-day fluctuations of subjective age, followed by stress and steps, whereas sunshine duration explained the largest share of variance in interindividual differences.

**Discussion and Implications:**

In our daily diary design, subjective age was most strongly related to self-reported affect as a psychological correlate. We, however, also found clear associations with objective data on daily steps and weather. Hence, our study contributes to contextualizing and understanding variations in subjective age in everyday life.

**Translational Significance**: Current research on the daily variability of subjective age primarily focuses on psychological predictors such as affect. In our daily diary study, we were able to show that behavioral factors such as daily steps also play a role in explaining variations in subjective age. In case light physical activity and mobility can be established as robust predictors of subjective aging, starting points for interventions would exist.

Subjective age—also referred to as felt age—is an established construct in aging psychology that has been linked to central developmental outcomes like control beliefs (Infurna et al., 2010), cognitive performance (Stephan et al., 2016), and health, including objective indicators such as biomarker profiles or longevity (Kotter-Grühn et al., 2009; Thyagarajan et al., 2019; Westerhof et al., 2023). Results from these larger cross-sectional and macro-longitudinal studies focus on lifespan development and approach subjective age as a relatively stable, *trait-like* construct, which is assumed to be part of an aging individual’s identity and self-concept (Dutt et al., 2018). However, a growing body of research has explored the *state-like* aspect of subjective age in experimental designs (e.g., Dutt & Wahl, 2017; for an overview, see Wahl & Kornadt, 2022) and micro-longitudinal studies (e.g., Kornadt et al., 2022).

Overall, micro-longitudinal studies demonstrated that subjective age varies considerably on a day-to-day basis. In the works of Kotter-Grühn et al. (2015) and Bellingtier et al. (2017), which followed *N* = 43 older U.S. American adults (age range: 60–96 years) with an 8-day diary survey, 23% of variance in subjective age was intraindividual. Comparable proportions (25% and 27%) could be attributed to within-person variation in the analyses of Kornadt et al. (2022, 2021) by drawing on subsamples (*N* = 170 and *N* = 154, age range: 66–90 years) from the German EMIL study (“Emotional Reactivity and Regulation in Old Age”) with a maximum of 32 assessments over 1 week. In a larger data set from Israel (Segel-Karpas et al., 2022; *N* = 334, age range 30–90 years), 49% of within-person variance in subjective age was reported over an observation period of 14 days.

## Theoretical Considerations: Processes of Anchoring and Adjusting

Such everyday variation in subjective age is assumed to be rooted in diverse everyday experiences. Theoretical work in this realm (Hughes & Touron, 2021; Montepare, 2009) states that subjective age varies based on proximal reference points (e.g., physical or interpersonal age markers, but also historic or normative markers), which can be one-time events such as retirement, but in particular repeated aging experiences in everyday life (Miche et al., 2014). These proximal reference points are then evaluated and reflected upon against stable developmental models (e.g., “feeling tired and not being able to go for a walk are part of being old”). Hence, the age an individual feels is assumed to be the subject of ongoing adjustment. Therefore, following a more contextual approach of subjective aging as proposed by Hughes and Touron (2021), we aimed to address diverse predictors from daily life to better understand the complexity surrounding variations in subjective age.

## Previous Research: Everyday Psychological Correlates of Subjective Age

Earlier research on everyday variation in subjective age has mostly focused on psychological correlates such as stress and affective mood. In the diary studies cited earlier, participants felt older on days with higher-than-average stress, negative affect, and depressive symptoms (Bellingtier et al., 2017; Kotter-Grühn et al., 2015; Segel-Karpas et al., 2022). In the experimental study conducted by Dutt et al. (2017), the induction of sad mood via both music and text leads to an older subjective age. Likewise, preceding stress—assessed by self-report and salivary cortisol levels—predicted an older subjective age in a study by Kornadt et al. (2022), but subjective age did not predict momentary variability in stress and vice versa. However, aside from psychological factors, research on behavioral and environmental factors that might further explain everyday variation in subjective age is scarce. A simultaneous investigation of more diverse correlates should help in understanding what exactly drives everyday variation in subjective age and as such, the more precise formation and adjustment processes behind subjective age. In the long run, such micro-longitudinal analyses may, in turn, help inform macro-longitudinal research and interventions targeting the crucial impact of subjective age on various indicators of health and survival (e.g., Westerhof et al., 2023).

## Physical Activity and Sleep as an Everyday Behavioral Predictor of Subjective Age?

Apart from psychological correlates, everyday behaviors, particularly health behaviors, may inform an individual about capabilities and limitations and as such constitute aging experiences. Regarding health behaviors, sleep problems and physical inactivity are both highly prevalent among older adults (Hallal et al., 2012; Jaussent et al., 2011) and likely serve as recurrent age markers. For example, being physically active as an older adult should go along with feelings of being physically fit/strong and perceiving the own body as competent—both have been linked to a younger subjective age (Caudroit et al., 2012; Montepare, 2006; Stephan et al., 2013, 2020), but has not been investigated within an everyday setting. In an attempt to differentiate between different levels of intensity, we aimed to apply *daily step counts* as a general indicator for mobility and mainly light physical activity and *minutes of moderate-to-vigorous physical activity* as indicators for more intense activities.

Sleep difficulties have been linked to various adverse mental and physical health outcomes as well as lower cognitive functioning among older adults (Cavuoto et al., 2016; Goldman et al., 2008; Hall et al., 2015; Magee et al., 2011). Poor sleep itself might be interpreted as a marker of old age or it may elicit other negative experiences the following day (e.g., memory difficulties, fatigue) that might be interpreted as such and lead to an older subjective age. In the past, only four studies have linked self-reported sleep measures to subjective age with mixed findings, and daily diary designs are missing to the best of our knowledge. Stephan et al. (2017) found that subjective age was a salient predictor of poor sleep quality beyond chronological age across three large U.S. surveys (Midlife in the United States Study, Health and Retirement Study, National Health, and Aging Trends Study). Data from a population-based Korean study (Yoon et al., 2023) indicated that a higher subjective age was associated with lower sleep quality, but only among older women. Sabatini et al. (2022) reported that lower sleep quality and shorter subjective sleep duration were related to higher awareness of negative age-related change (see Diehl & Wahl, 2010), but relations with subjective age were negligible. Very recently, Balter and Axelsson (2024) found in a cross-sectional study (age range: 18–70 years) that both the number of days with insufficient sleep in the last month as well as the level of sleepiness were associated with a higher subjective age in relation to calendar age. To better understand the interplay between everyday health behaviors and subjective age, we thus investigated physical activity and self-reported sleep quality as everyday behavioral correlates.

## Weather Conditions as an Environmental Predictor of Subjective Age

Similar to behavioral correlates, everyday correlates of subjective age that lie outside of the individual have rarely been investigated in past research. One exception is the work by Goecke and Kunze (2020), which shows that negative work events account for everyday variation in subjective age. Following the call to contextualize psychological aging research (Hughes & Touron, 2021; Neupert & Bellingtier, 2022; Wahl & Gerstorf, 2018), we aimed to include weather conditions as a highly salient environmental aspect of older individuals’ lives, also tied in with our proposed psychological and behavioral correlates. Weather conditions have been shown to profoundly shape the lives of older adults—for example, by affecting their physical activity levels, time out of home, participation in society, as well as affective state (Klimek et al., 2022; Kööts et al., 2011; Petersen et al., 2015; Wu et al., 2017). Sunshine duration in particular may lead to younger felt ages via all these pathways, but in particular a higher out-of-home activity and positive affective pathways.

## Research Aims and Hypotheses

Our overall research aim was to acquire a more profound understanding of subjective age’s everyday variation and covariation with diverse, but potentially interwoven, psychological, behavioral, and environmental aspects of everyday life. We aimed to address the population of healthy older adults in “third age” (i.e., from retirement up to an age of approximately 80 years; see Baltes & Smith, 2003) and to extend the observation period (i.e., number of days sampled) in comparison to previous studies. Specifically, we sampled data from up to 21 days spaced over 5–6 weeks. Compared to previous studies targeting everyday subjective age, this longer observation period allowed to capture more heterogeneous subjective aging experiences (Kotter-Grühn et al., 2015) as well as sufficient intraindividual variability in psychological, behavioral, and environmental variables, which are likely to fluctuate with different intensity and on different time scales.

Using these more extensive data, we first aimed to replicate existing findings showing considerable intraindividual variability in subjective age and its meaningful relationships with stress and affective mood as psychological correlates. Second, we aimed to expand cross-sectional and long-term longitudinal relationships between physical activity, sleep, and subjective age by investigating them in short-term intervals. Specifically, we expected a higher number of daily steps, a higher amount of moderate-to-vigorous activity, and better self-reported sleep quality to be related to a younger daily subjective age. Third, we aimed to explore the impact of daily sunshine duration as a salient environmental factor and expected individuals to feel younger on sunnier days.

## Method

### Recruitment and Sample

The present analyses are based on data of the ActiveAge project, a physical activity intervention study for retired adults aged 60+ who exhibit low physical activity levels and intend to increase their physical activity (Schmidt et al., 2022). Ethical approval was obtained from the ethics commission of the Faculty of Behavioural and Cultural Studies at Heidelberg University.

Participants were recruited via flyers and newspaper articles in the Rhine-Neckar Metropolitan Region in Germany throughout the year 2017. They did not receive monetary compensation but were offered feedback on their physical activity and the chance of winning an activity tracker. A total of 135 older adults expressed interest in participation and were screened via telephone by trained scientific staff based on the following inclusion criteria: (1) retired or working less than 10 hr/week (including voluntary work), and (2) no or only very low levels of physical activity. Moreover, the following exclusion criteria were applied: (1) severe functional limitations, acute pain, or chronic conditions preventing physical activity; (2) severe visual impairments; (3) acute depressive episode; (4) severe cognitive impairment; and (5) prior experience with activity trackers. Fifty individuals did not meet the inclusion criteria or fulfilled one of the exclusion criteria, and five dropped out during the first week due to the death or illness of a close other.

The final sample consisted of *N* = 80 retired individuals aged 59–76 years (*M* = 67.03, standard deviation [*SD*] = 3.97). Fifty-nine percent of the sample were women, 63% were married, 12% widowed, and 25% divorced, separated, or single. The sample’s education was above the population average (*M* = 12.05 years of schooling, *SD* = 2.15). Participants rated their own health mainly as good or very good (*M* = 2.92, *SD* = 0.65) on a scale ranging from 1 *(excellent)* to 5 *(bad)* (Morfeld et al., 2011). Informed consent was obtained from all participants. More information on background characteristics, study aims, and findings not in the focus of the present analyses can be found in Schmidt et al. (2022).

### Procedure

The ActiveAge study was designed as a pre–post physical activity intervention without a control group and was mainly based on monitoring, feedback, and goal setting as behavior change techniques (Marques et al., 2023). The study included a baseline questionnaire (T0; online or paper–pencil) and three personal standardized interviews (T1–T3) that were each followed by 7-day diary periods (paper–pencil) alongside physical activity measurement. T1 and the first week of measurement were immediately followed by T2 and the second measurement week, whereas a break of 2 weeks (in some cases 3 weeks due to scheduling problems for the participants) separated the second measurement period and T3. Hence, diaries should be kept for 21 days, within an average scope of 5–6 weeks as the study duration. Participants kept diaries for an average of *M* = 20.13 days (*SD* = 3.73). Out of initially 1,610 daily assessments, 122 had to be excluded due to missing subjective age. Further 205 assessments were excluded due to invalid data on physical activity. Altogether, there were 1,283 assessments available from 80 individuals: *M* = 15.35, *SD* = 3.84.

### Measures

#### Daily diary variables

Participants were asked to complete their diary every evening and to answer questions on subjective age, stress, affect, and sleep duration.

For subjective age, a proportional discrepancy score was calculated following Rubin and Berntsen (2006): We subtracted chronological age from the answer to the question “All in all, how old did you feel today?” (subjective age) and divided the result by chronological age. Five values were identified as extreme values (>*M* + 3*SD*) and excluded from analyses. After multiplying the respective proportional discrepancy with 100 for reasons of clarity, a score of −8.0 would indicate feeling 8% younger than one actually is.

Perceived stress was assessed with one item asking participants to rate their subjective stress level on the respective day on a visual analogous scale between 0 *(not stressed at all)* and 100 *(totally stressed)* (Lesage & Berjot, 2011).

Affective mood was measured with the two-item valence subscale of a short mood scale that has proven to be reliable, but also sensitive to change in measurement burst designs (Wilhelm & Schoebi, 2007). The two mood items were answered on bipolar scales ranging from negative to positive poles, that is, 0 (*discontent*) to 6 (*content*) and 0 (*unwell*) to 6 (*well*). The two items were averaged.

Sleep quality was assessed with a single item (“How well did you sleep last night?”) based on the Pittsburgh Sleep Quality Index (Buysse et al., 1989) on a scale from 1 (*very poorly*) to 7 (*very well*).

#### Physical activity

For measuring physical activity, the wrist-worn, commercially available activity tracker Fitbit Charge HR (Fibit, Inc., San Francisco, CA) was used. For privacy reasons and in order to not exclude older adults without a smartphone, we created e-mail aliases and pseudonymous accounts that were not connected to the mobile Fitbit app. As a first indicator for (mainly) light physical activity and mobility, we used step counts, and as a second indicator, we used minutes spent with moderate-to-vigorous physical activity (MVPA). The Fitbit has performed well in measuring step counts and MVPA in earlier research (e.g., Paul et al., 2015) and in our own pilot study where participant compliance and device performance were very satisfactory (Schmidt, Gabrian et al., 2018). We collected information on compliance via self-reports on nonwearing periods in the diaries. Two hundred and five invalid days occurred especially due to insufficient wearing times (e.g., forgot to recharge), difficulties of the device in specific circumstances (e.g., motorcycling), and technical errors. To ease interpretation, the number of steps was divided by 1,000 for the multilevel regression analyses.

#### Weather data

The weather data stemmed from the local weather station in Mannheim, Germany. Sunshine was measured in hours per day. Average sunshine duration over the assessment period ranged from 0.56–9.99 hr/day, clearly reflecting the year-round assessment.

#### Covariates

As our main interest lies in intraindividual associations, we applied interindividual covariates sparsely and only focused on chronological age and sex (0 = women, 1 = men). As an intraindividual covariate, we accounted for the passing of time by entering the respective diary day, starting with Day 0 and amounting to Day 20. The passing of time hereby serves as a proxy for potential intervention effects associated with the pre–post physical activity intervention design. Other control variables that were derived from the original intervention study, such as measurement week or study group, were also tested in earlier analyses but did not reveal additional effects.

### Data Analysis

As the single measurement points (days) were nested within individuals, we used multilevel modeling to account for effects on the level of the individual (Level 2) and effects on the level of intraindividual and day-to-day dynamics (Level 1). We entered age and sex as covariates on Level 2 and modeled all other variables on Level 1. All predictor variables and covariates except time and sex were centered around the grand mean. We started with Model 1, where we built a random-intercept model (allowing for an individual-specific mean in subjective age over the assessment period) and entered all covariates. For Models 2–7, each everyday predictor variable (stress, affect, MVPA, steps, sleep quality, and sunshine duration) was added as a single predictor. By doing so, we determined the strength of each of the six correlates and could determine whether they explained variance on Level 2 (more stable tendencies in subjective age) or Level 1 (intraindividual, everyday dynamics in subjective age). Model 2 focuses on stress, Model 3 on affect, Model 4 on physical activity, Model 5 on steps, Model 6 on sleep quality, and Model 7 on sunshine. Model 8 finally includes all covariates and predictors on Level 1 and Level 2. The models were thus based on the equation $SA_{ti}=\beta_{0}+\beta_{j}C_{\left(\;j\right)i}+\beta_{k}P_{\left(k\right)ti}+u_{0i}$, where $SA_{ti}$ denotes the subjective age for individual *i* at time *t*, $\beta_{0}$ denotes the sample mean, and $u_{0i}$ denotes the variance of the mean across individuals. The term $\beta_{j}C_{\left(\;j\right)i}$ applies to Level 2 predictors *j*, and the term $\beta_{k}P_{\left(k\right)ti}$ applies to all intraindividual predictor variables *k*.

Due to the relatively high homogeneity of the sample (i.e., less Level-2 variance than Level-1 variance, see Table 1), all daily variables were entered on Level 1 and combined Level-2 variance and Level-1 variance for the main analyses. In Supplementary Table 1, analyses are presented with separated Level-2 and Level-1 variance.

**Table 1.** Descriptives and Bivariate Intercorrelations of the Study Variables.

Variable	Mea*n*	*SD*_inter_	*SD*_intra_	ICC	1	2	3	4	5	6	7	8	9
1. Subjective age	−8.63	6.48	5.48	0.58		0.21	−0.21	−0.08	−0.09	−0.07	**−0.25**	0.01	−0.04
2. Stress	27.09	14.94	16.03	0.46	**0.21**		**−0.47**	−0.22	−0.19	**−0.26**	−0.11	**0.28**	−0.17
3. Mood	3.96	0.69	1.06	0.30	**−0.48**	**−0.26**		0.21	0.16	**0.39**	0.06	**−0.22**	−0.04
4. MVPA	40.45	26.66	38.70	0.32	**−0.12**	**−0.11**	**0.18**		**0.58**	0.03	0.02	−0.08	0.03
5. Steps	10,954.44	3,078.59	3,722.77	0.41	**−0.21**	**−0.12**	**0.24**	**0.66**		0.05	−0.02	−0.06	−0.10
6. Sleep quality	5.21	0.70	1.10	0.29	**−0.08**	**−0.21**	**0.13**	**0.07**	**0.06**		−0.14	0.06	−0.03
7. Sunshine duration (h)	3.54	1.88	3.49	0.22	**−0.07**	−0.05	**0.08**	0.05	**0.07**	−0.03		0.03	0.07
8. Age	67.03	3.97											**0.26**
9. Sex													

*Notes*: ICC = intraclass correlation; MVPA = moderate-to-vigorous physical activity; *SD* = standard deviation. Subjective age is operationalized as a proportional difference score. Means, interindividual and intraindividual standard deviations, as well as ICCs are reported on the left side of the table. Bivariate Intraindividual correlations (Level 1) are reported below the diagonal, interindividual correlations (Level 2) are reported above the diagonal. Significant correlations (*p* < .05) are printed bold.

## Results

### Descriptives

Table 1 depicts means, inter- and intraindividual *SD*s, intraclass correlations (ICCs), as well as inter- and intraindividual correlations. Across the 21 days, participants felt on average 8.6% younger than their chronological age. They showed high levels of physical activity with an average of 10,954 steps and 40 min of daily MVPA. On a bivariate level, the only significant interindividual correlate of subjective age was hours of sunshine. Thus, individuals who participated during sunnier periods felt younger on average. Intraindividually, an older subjective age occurred on days where individuals reported more stress, worse mood, and worse sleep quality, as well as on days on which less MVPA, fewer steps, and fewer hours of sunshine were measured. All everyday study variables displayed considerable amounts of intraindividual variance, with shares between 44% (subjective age) and 78% (hours of sunshine) of the whole variance.

### Does Subjective Age Fluctuate Within Short Time Frames?

**Figure 1.**
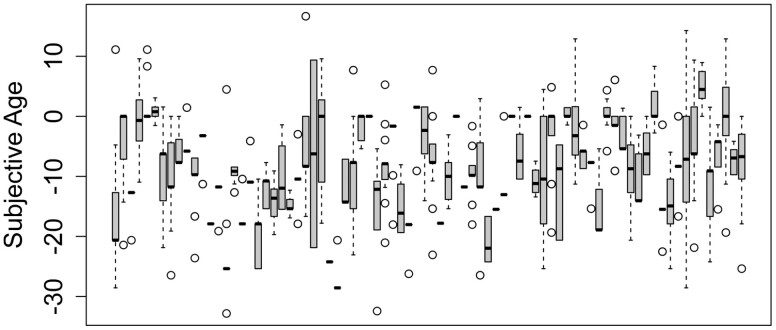
Boxplots with intraindividual variation in subjective age for each of the 80 participants.

Subjective age fluctuated considerably within the 21 diary days and 5 weeks of investigation. With an ICC of 0.56, 44% of the overall variance in subjective age was intraindividual (Table 1). The intraindividual variability of subjective age over the diary period is depicted in Figure 1. The intraindividual *SD* (*M* = 5.79) ranged from 0 to 13.99. In some of the models (see Table 2), there was a positive time trend in subjective age, indicating that individuals felt slightly older, the longer they participated in the study.

**Table 2.** Subjective Age in Relation to Stress, Affect, MVPA, Steps, Sleep Quality, and Hours of Sunshine

Variable	Model 1	Model 2	Model 3	Model 4	Model 5	Model 6	Model 7	Model 8
Intercept	**−8.99** (1.03)	**−9.34** (1.01)	**−8.83** (1.00)	**−9.25** (1.03)	**−9.15** (1.03)	**−9.00** (1.08)	**−8.88** (1.02)	**−9.03** (1.08)
Day	**0.06** (0.03)	**0.07** (0.03)	0.05 (0.02)	**0.06** (0.03)	**0.07** (0.03)	**0.06** (0.03)	**0.06** (0.03)	0.04 (0.03)
Age	0.04 (0.20)	−0.06 (0.19)	−0.04 (0.19)	0.05 (0.20)	0.02 (0.20)	0.06 (0.20)	0.04 (0.20)	−0.08 (0.20)
Sex	−0.64 (1.59)	−0.02 (1.56)	−0.92 (1.56)	−0.38 (1.59)	−0.53 (1.60)	−0.62 (1.59)	−0.60 (1.58)	−0.49 (1.57)
Stress		**0.08** (0.01)						**0.06** (0.06)
Mood			**−2.44** (0.14)					**−2.31** (0.16)
MVPA				**−0.01** (0.00)				0.01 (0.01)
Steps					**−0.30** (0.04)			**−0.19** (006)
Sleep quality						**−0.43** (0.15)		−0.03 (0.15)
Sunshine							**−0.11** (0.05)	−0.05 (0.05)
*R*²_between_ (% explained)	43.56	41.44 (5%)	41.87 (4%)	43.38 (0%)	44.08 (0%)	43.24 (1%)	42.78 (1%)	41.55 (5%)
*R*²_within_ (% explained)	29.91	29.31 (2%)	23.56 (21%)	29.72 (1%)	28.70 (4%)	30.26 (0%)	29.82 (0%)	24.13 (19%)
*R*² (% explained)	73.47	70.75 (4%)	65.43 (11%)	73.10 (1%)	72.78 (0%)	73.50 (0%)	72.60 (1%)	65.68 (11%)

*Notes*: MVPA = moderate-to-vigorous physical activity. Subjective age is operationalized as a proportional difference score and is predicted by multilevel models. Model 1 includes the Level-1 covariate day and the Level-2 covariates age and sex. Models 2–8 include Level-1 predictors, which combine Level-2 and Level-1 variance. Unstandardized coefficients are given together with standard errors in parentheses. Significant parameters (*p* <* *.05) are printed bold.

### Stress and Affective Mood as Psychological Correlates of Subjective Age

In Table 2, Models 2 and 3 indicate that both stress and affective mood were significantly related to subjective age. In comparison to a model controlled for day, age, and sex, stress explained 2% of variance on the intraindividual level and 5% of variance on the interindividual level. This indicates that individuals who felt more stressed, on average, also felt older on average. Additionally, individuals felt younger on days on which they felt less stressed than usual. Daily mood explained 21% of variance on the intraindividual level and 4% of variance on the interindividual level, indicating a strong relationship between daily affect and daily subjective age; individuals felt younger in particular on days with a better mood. The associations held even when all other predictors were entered into Model 8. Stress and mood could thus be replicated as everyday correlates of subjective age.

### Physical Activity and Sleep as Behavioral Correlates of Subjective Age

In Table 2, Models 4–6 indicate that MVPA, steps, and sleep quality were significantly related to subjective age. MVPA and steps explained 1% and 4% of variance on the intraindividual level, respectively, indicating that individuals felt younger on days on which they were relatively more physically active or mobile. Specifically, in comparison to days with absolutely no physical activity and mobility, individuals would feel around 2.1% younger when they were active for 2 hr and would feel around 3.0% younger after taking 10,000 steps. Sleep quality explained 1% of variance on the interindividual level indicating that individuals who reported better sleep on average would also report a younger subjective age on average. The difference between sleeping *very poorly* versus *very well* would hereby amount to feeling around 2.6% younger. Despite significant effects, the variance explanation for MVPA and sleep quality was low, and some variance components even increased in size. This likely indicates the presence of random slopes, meaning that the relationship between MVPA and subjective age as well as sleep quality and subjective age differed from individual to individual. As soon as other predictors were entered into Model 8, only steps remained as a significant predictor. All unstandardized effects (for MVPA, steps, and sleep quality) clearly decreased in size, which suggests that the other predictors, in particular stress and affective mood might work as mediators: On less active days and after nights with bad sleep, individuals might have felt more stressed and in a worse mood, which would then again explain older subjective ages.

### Sunshine Duration as an Environmental Correlate of Subjective Age

In the single-predictor model (Table 2, Model 7), longer sunshine duration was related to a lower subjective age, however, only explaining 1% of variance on the interindividual level, *b* = −0.11. This was in contrast with the interindividual correlation of *r* = 0.25 we found in Table 1, which would indicate a variance explanation of 6% on the interindividual level. One possibility for this result could be that inter- and intraindividual effects of sunshine duration were overlapping in Table 2. In Supplementary Table 1, we disentangled the effects of average sunshine duration across the assessment period (Level 2 predictor) and intraindividual deviations from this average (Level-1 predictor). When doing so, effects were indeed larger, with *b* = −0.78, standard error (*SE*) = 0.35, *p* = .030, and 5% of variance explanation on the interindividual level, and *b* = −0.13, *SE *= 0.05, *p* = .030, and 0% of variance explanation on the intraindividual level (overall variance explanation was 3%). Following these results, individuals were feeling approximately 7.4% younger when the sun was shining the maximum average of 10 hr/day compared to the minimum average of 0.5 hr. When other predictors were entered into the model (Table 2, Model 8), the overall effect of sunshine duration vanished. However, the purely interindividual effect of the supplement remained significant. Taken together, daily fluctuations in sunshine duration did not seem particularly consequential for subjective age. However, individuals who participated during sunnier periods (Western European summer) felt much younger on average than individuals participating during cloudy periods (Western European winter).

## Discussion

Using a daily diary design, we aimed to investigate the potential explanatory power of physical activity and sleep as behavioral factors, and sunshine duration as an environmental factor for daily variations in subjective age beyond more established psychological correlates in this research area. In the first step, we were able to confirm considerable variability in subjective age and to replicate findings supporting a significant relation between everyday stress and affective mood (Kotter-Grühn et al., 2015) across a longer observational period of 3 weeks. Going beyond, physical activity, sleep quality, and weather conditions also explained portions of subjective age variance. Of note, affect still explained most of the variance in day-to-day fluctuations of subjective age.

The fluctuations of subjective age in this study clearly substantiate its proposed state component (see also Dutt & Wahl, 2017). The share of intraindividual variance in subjective age was larger than in Kotter-Grühn et al. (2015) and Kornadt et al. (2021), but comparable to Segel-Karpas et al. (2022) who—as in the present study—sampled subjective age for a longer period of time. Potentially, our results also point to early retirement as a period of pronounced fluctuations in subjective age.

### Intraindividual Variability in Subjective Age and its Relation to Affect

The everyday variation in subjective age is non-arbitrary as it covaries with other variables—most strongly with affect. Whereas earlier work was able to show short-term relations between subjective age and negative but not positive affect (Bellingtier et al., 2017), we were able to establish the relation between subjective age and valence of affect by using a bipolar scale. Participants felt younger on days on which they were well rather than unwell, and content rather than discontent. Affect might reflect the count of negative (aging) experiences during the day (such as social situations and health problems) and be a mediating source of fluctuations in subjective age. Also, individuals in a negative mood might concentrate stronger on negative (aging) experiences and therefore feel older (Dutt & Wahl, 2017; Miche et al., 2014). Similarly, affect might have an impact on how individuals deal and cope with everyday (aging) experiences.

### Additional Everyday Correlates of Subjective Age

In addition to affect, stress and steps accounted for small shares of everyday variance in subjective age. Relations were as hypothesized. Individuals felt older on stressful days and during stressful periods—although the effect of stress was considerably smaller than the one found by Kotter-Grühn et al. (2015). Participants also felt younger on days they took more steps, whereas the association between subjective age and more strenuous physical activity (MVPA) disappeared when other predictors (specifically, steps) were entered into the model. In contrast to MVPA, steps per day might include time out-of-home, mobility, and general participation in societal life, which may crucially relate to subjective age. However, the fact that we included only participants with previously (very) low MVPA levels, who then exhibited higher-than-usual MVPA throughout the entire measurement period, should be kept in mind. In the original intervention study (Schmidt et al., 2022), there was already a significant increase in physical activity between the first cross-sectional survey and the measurement period used here; the mean change in physical activity within the measurement period of the present study was correspondingly very small. This selection might have restricted variance in MVPA leading to biased associations with subjective age. Future research, therefore, needs to clarify whether physical activity or related factors such as mobility are actually relevant for subjective age.

Self-reported sleep quality was a significant correlate of subjective age only in the single-predictor model. Individuals felt younger after a night of good sleep. This association did, however, not persist when other predictors were entered into the model; the effect of sleep quality was most likely explained by stress and affect, with which sleep quality showed significant relations on the bivariate level. Our findings strongly relate to the study of Sabatini et al. (2022), whose linear regression models indicated that poorer sleep quality was significantly associated with a higher subjective age (*R*² = 1.0%), but after adjusting for covariates, the associations became weaker (*R*² = 0.02%). Behavioral correlates such as sleep, physical activity, and steps are potentially modifiable predictors of subjective age. Meta-analyses point to at least small positive effects of physical activity and sleep interventions in community-dwelling older adults (Chase, 2015). Nonpharmacological intervention programs incorporating physical activity to improve sleep quality in older adults were particularly promising (Sella et al., 2023; Vanderlinden et al., 2020). If future research can establish physical activity, steps and sleep as robust correlates of subjective aging, even if mediated by psychological variables such as affective mood, starting points for interventions would hence exist.

Finally, sunshine duration had a negligible intraindividual effect, which vanished as soon as other variables (e.g., affect, steps) were entered into the model. Sunshine duration was, however, consistently related to subjective age on the interindividual level, suggesting that individuals participating during sunnier periods or seasons felt several years younger than those participating during cloudy or darker seasons. This does not come as a surprise because weather conditions shape the everyday life and aging experiences of older adults to a considerable degree (Hoppmann et al., 2017; Kööts et al., 2011; Petersen et al., 2015). However, weather has so far not been investigated in the context of subjective age. Naturally, weather may have a different impact on other population groups. For example, individuals of various ages and health states might be affected very differently by their environment (Wahl & Gerstorf, 2018). As weather may constitute an influential, environmental predictor with clear causality (i.e., individuals’ subjective ages cannot affect weather), further research could contribute largely to the understanding of (explanatory) mechanisms behind subjective age. Our findings may stimulate new research with weather conditions as predictor variables as well as control variables. For example, in longitudinal study designs covering summer and winter periods, but also cross-sectional designs that take place during a time of weather change, weather might be a source for otherwise unexplainable interindividual variation. Furthermore, future research needs to disentangle the direct effects of weather on subjective age from mediating effects, for example, social and physical activities or mood that change with weather conditions.

The theoretical underpinning of our study (Hughes & Touron, 2021; Montepare, 2009) states that proximal aging experiences are continuously evaluated against more stable conceptions of development (i.e., what it means to be old) and lead to fluctuations in subjective age. Our findings support this theory as subjective age fluctuated and was meaningfully related to variables that directly or indirectly reflect aging experiences. However, apart from weather (and sleep quality which was explicitly sampled in regard to the previous night), our statistical tools did not allow us to test for causal or time-ordered mechanisms. Hence, the relations between subjective age and stress, affect, steps, and physical activity might be reciprocal or in the opposing direction. For example, individuals might be more physically active as a consequence of feeling younger.

### Strengths, Limitations, and Future Research

The statistical analysis of the current study focused on cross-sectional associations and does not allow for claims of causality. Time-ordered analyses and experimental designs (like Dutt & Wahl, 2017; Stephan et al., 2013) should investigate the directionality in the relation between everyday experiences and subjective age. Second, due to the specific target group of the original ActiveAge intervention (newly retired older adults without chronic conditions preventing physical activity), our sample was rather homogeneous. This may explain why less variance was explained on the interindividual level and may limit our findings’ generalizability. More diverse samples would likely be needed to study moderators and particularly, whether certain developmental ideas (e.g., future expectations, views on aging) may affect the role of everyday predictors of subjective age (see Montepare, 2009). In this regard, it would also be interesting to focus more strongly on random slopes (i.e., individual-specific effects), for which we found statistical indications in our analyses. Third, there were shortcomings and strengths in the study design and assessments. Sleep quality was captured via self-report at the end of the day, which might be more prone to recall bias than immediate morning diaries. Future research may use additional objective sleep assessments, for example, wearables able to measure a broader range of sleep variables such as sleep duration, times awake, time spent in each of the sleep stages, or sleep efficiency. The objective assessment of physical activity namely comes with many advantages: It is more valid, accurate, and reliable than self-reports of physical activity (Prince et al., 2020), and less influenced by social desirability and demand effects. However, the objective assessment also resulted in missings and exclusion of certain data points. Fourth, we chose sunshine duration as a conceptually meaningful indicator of weather and environment in the specific setting of our study. Depending on geographical and historical contexts, other environmental indicators (e.g., maximum or minimum temperatures, climate events) may be able to better capture the variations and extremes individuals are facing in their objective and subjective aging process.

## Conclusion

The diary study covered 21 days and allowed for an in-depth replication of prior findings that subjective age varies considerably in everyday life. We were able to identify a number of everyday correlates of subjective age. The psychological correlates, stress and affective mood, were hereby the strongest. The number of steps served as a robust behavioral correlate of subjective age. In contrast, the effects of more strenuous physical activity and sleep quality were small and seemed to be explained by the other predictors. Lastly, our year-round assessment and the spacing of the diary periods over 5–6 weeks allowed for the first investigation of the effect of weather conditions as an environmental predictor of (daily) subjective age, with individuals reporting younger subjective ages during sunnier periods of the year. With objective data on physical activity and weather, our study contributes to contextualizing and understanding subjective age in everyday life.

## Supplementary Material

igae067_suppl_Supplementary_Tables

## Data Availability

The conducted research was not preregistered. Data and analytic code relevant for the reported analyses as well as details on study design and measures are available from the corresponding author upon reasonable request.
