# Supplementary material for: Feeling Younger on Active Summer Days? On the Interplay of Behavioral and Environmental Factors With Day-to-Day Variability in Subjective Age
Source: Innov Aging. 2024 Jul 17;8(8):igae067. doi: 10.1093/geroni/igae067 (PMC11319866; doi:10.1093/geroni/igae067)
Supplement: igae067_suppl_Supplementary_Tables [file igae067_suppl_supplementary_tables.docx]

***Innovation in Aging* Supplementary Material: Schmidt et al. Feeling younger on active summer days? On the interplay of behavioral and environmental factors with day-to-day variability in subjective age.**

| **Supplementary Table 1.** Subjective age in relation to stress, affect, MVPA, steps, sleep quality, and hours of sunshine with separated Level 2 and Level 1 variance. | | | | | | | | |
| --- | --- | --- | --- | --- | --- | --- | --- | --- |
|  | Model 1 | Model 2 | Model 3 | Model 4 | Model 5 | Model 6 | Model 7 | Model 8 |
|  |  |  |  |  |  |  |  |  |
| Intercept | **-8.99** (1.03) | **-9.44** (1.03) | **-8.75** (1.00) | **-9.26** (1.04) | **-9.13** (1.04) | **-9.00** (1.03) | **-8.88** (1.02) | **-9.53** (1.07) |
|  |  |  |  |  |  |  |  |  |
| Day | **0.06** (0.03) | **0.07** (0.03) | 0.05 (0.02) | **0.06** (0.03) | **0.07** (0.03) | **0.06** (0.03) | 0.05 (0.03) | 0.04 (0.03) |
| Age | 0.04 (0.20) | -0.08 (0.21) | -0.02 (0.20) | 0.05 (0.20) | 0.03 (0.20) | 0.06 (0.20) | 0.05 (0.19) | -0.05 (0.21) |
| Sex | -0.64 (1.59) | 0.14 (1.62) | -0.94 (1.56) | -0.38 (1.60) | -0.56 (1.60) | -0.64 (1.60) | -0.57 (1.56) | -0.06 (1.64) |
|  |  |  |  |  |  |  |  |  |
| Stress_Average_ |  | 0.10 (0.05) |  |  |  |  |  | 0.06 (0.06) |
| Stress_Daily_ |  | **0.07** (0.01) |  |  |  |  |  | **0.03** (0.01) |
|  |  |  |  |  |  |  |  |  |
| Affect_Average_ |  |  | **-2.00** (1.00) |  |  |  |  | -1.09 (1.24) |
| Affect_Daily_ |  |  | **-2.45** (0.14) |  |  |  |  | **-2.33** (0.16) |
|  |  |  |  |  |  |  |  |  |
| MVPA_Average_ |  |  |  | -0.02 (0.03) |  |  |  | 0.02 (0.03) |
| MVPA_Daily_ |  |  |  | **-0.02** (0.00) |  |  |  | 0.01 (0.01) |
|  |  |  |  |  |  |  |  |  |
| Steps_Average_ |  |  |  |  | -0.19 (0.24) |  |  | -0.14 (0.29) |
| Steps_Daily_ |  |  |  |  | **-0.30** (0.04) |  |  | **-0.19** (0.06) |
|  |  |  |  |  |  |  |  |  |
| Sleep quality_Average_ |  |  |  |  |  | -0.68 (0.98) |  | 0.13 (1.09) |
| Sleep quality_Daily_ |  |  |  |  |  | **-0.42** (0.15) |  | -0.04 (0.15) |
|  |  |  |  |  |  |  |  |  |
| Sunshine_Average_ |  |  |  |  |  |  | **-0.78** (0.35) | **-0.77** (0.38) |
| Sunshine_Daily_ |  |  |  |  |  |  | **-0.13** (0.05) | -0.04 (0.05) |
|  |  |  |  |  |  |  |  |  |
| R²_between_ (% explained) | 43.56 | 41.89 (3%) | 42.31 (3%) | 43.97 (0%) | 44.52 (0%) | 43.80 (0%) | 41.28 (5%) | 41.76 (4%) |
| R²_within_ (% explained) | 29.91 | 29.31 (2%) | 23.57 (21%) | 29.72 (1%) | 28.70 (4%) | 30.26 (0%) | 29.82 (0%) | 24.13 (19%) |
| R² (% explained) | 73.47 | 71.20 (3%) | 65.88 (10%) | 73.69 (0%) | 73.22 (0%) | 74.06 (0%) | 71.10 (3%) | 65.89 (10%) |

*Note.* Subjective age is predicted by multilevel models. Model 1 includes the Level-1 covariate day and the Level-2 covariates age and sex. Models 2 to 8 include daily predictors modelled as individual averages (Level 2; labelled as average) and intraindividual daily deviations from this average (labelled as daily). Unstandardized coefficients are given together with standard errors in parentheses. Significant parameters (*p* < .05) are printed bold.
